# Response to the Union’s statement on ongoing conflicts and impact on health services

**DOI:** 10.5588/ijtldopen.26.0044

**Published:** 2026-04-13

**Authors:** F. Ahmad Khan, R. Menzies, A. Ashesh, E. Bijker, S. Brode, E.J. Carter, E.C. Casas, J. Campbell, C. Denkinger, S. Deborggraeve, I. Evlampidou, J. Furin, M. Farhat, M.M.O. Farouk, F. Gafar, G. Günther, L. Guglielmetti, P. Isaakidis, F. Jouberton, N. Khambati, P.Y. Khan, U. Khan, R. Long, S. Law, A. Matteelli, D. Malden, I. Motta, M. Murray, T. Masini, M. Rich, R. Ruslami, C. Sepulcri, T. Sunyoto, S. Tunesi, E.B. Wong, A. Zaffagnini

**Affiliations:** 1 McGill International TB Centre, McGill University, Montreal, Canada;; 2 Patna, India;; 3 Department of Paediatrics, University of Oxford, Oxford, UK;; 4 Department of Medicine, Division of Respirology, University of Toronto, Toronto, Canada;; 5 School of Medicine, Brown University, USA;; 6 Southern Africa Medical Unit, Médecins Sans Frontières (MSF), Cape Town, South Africa;; 7 Heidelberg University Hospital, Heidelberg, Germany;; 8 Operational Centre Brussels, Médecins Sans Frontières (MSF), Brussels, Belgium ;; 9 Luxembourg Operational Research and Epidemiology Support Unit, Operational Centre Brussels, Médecins Sans Frontières (MSF), Brussels, Belgium;; 10 Harvard Medical School, Department of Global Health and Social Medicine, Boston, USA;; 11 Pulmonary and Critical Care Physician, Boston, USA;; 12 Operational Directorate Western and Central Africa, Médecins Sans Frontières (MSF), Niamey, Niger;; 13 Department of Biomedical Sciences, Faculty of Medicine, Universitas Padjadjaran, Bandung, Indonesia;; 14 Department of Pulmonology, Allergology and Clinical Immunology, Inselspital, Bern University Hospital, Bern, Switzerland;; 15 Department of Infectious, Tropical Diseases and Microbiology, IRCCS Sacro Cuore Don Calabria Hospital, Negrar di Valpolicella (Verona), Italy;; 16 Southern Africa Medical Unit, MSF, Cape Town, South Africa;; 17 International Office, Médecins Sans Frontières (MSF), Geneva, Switzerland;; 18 Department of Clinical Research, Faculty of Infectious and Tropical Diseases, London School of Hygiene & Tropical Medicine, London, UK;; 19 College of Health Sciences, Faculty of Medicine, University of Alberta, Edmonton, Canada;; 20 Clinic of Infectious and Tropical Diseases, Department of Clinical and Experimental Sciences, University of Brescia, Brescia, Italy;; 21 Medical Research Council Clinical Trials Unit at University College London, London, UK;; 22 Access Team, Medecins Sans Frontieres, Geneva, Switzerland;; 23 Camaiore (Lucca), Italy;; 24 Partners In Health, Boston, USA;; 25 Department of Health Sciences, University of Genova, Italy;; 26 Azienda Ospedaliero-Universitaria SS Antonio e Biagio e C. Arrigo, Alessandria, Italy;; 27 Division of Infectious Diseases, Heersink School of Medicine, University of Alabama, Birmingham, USA;; 28 Africa Health Research Institute, Durban, South Africa.

**Keywords:** healthcare workers, human rights, Geneva Conventions, genocide, Gaza

Dear Editor,

During the 2025 Union World Conference on Lung Health in Copenhagen, The Union Board of Directors took the opportunity to issue a statement regarding armed conflicts, calling ‘on all relevant actors, including those directly involved and those indirectly involved, but with influence on the events, to ensure adherence to the principles and articles of international law including the protection of civilians and healthcare workers, ensuring access to food, sanitation and medical supplies, and reconstruction of the health system.’^1^ The Union’s statement reflects a moral obligation of all organizations and individuals working in global and public health to call for the enforcement of international laws that protect human rights and dignity during times of war, established in direct response to the atrocities committed during the Holocaust and WWII. International human rights law is a cornerstone of the foundation upon which global health is built. Similarly, global health practitioners and institutions must support the investigations and rulings of the International Criminal Court and the International Court of Justice—institutions that exist to protect civilians, uphold humanitarian principles, and ensure accountability for violations that directly undermine global health.

Unfortunately, there are many sites in the world, such as Myanmar, Palestine, Sudan, and Ukraine, where human rights are being violated and the principles and articles of international law are not being upheld at this time. To save lives urgently, we believe specific advocacy directed to each particular situation is needed. As authors of this article, we believe moral obligations compel global and public health organizations to specifically advocate to end what we consider as genocide and healthocide in Gaza, Palestine.^2–7^ This is not to diminish other conflicts, but to address the specific circumstances that make silence about Gaza particularly problematic for our field. In Gaza, relentless bombardment has created an extreme and ongoing threat to respiratory health,^8^ while healthcare workers and facilities have been systematically targeted – over 1,500 health workers killed, others detained, and hospitals and ambulances repeatedly attacked.^9–11^ Israel’s destruction of homes and the near-total collapse of water, sanitation, and health infrastructure has made basic healthcare and disease prevention impossible.^12^ These actions have been declared a genocide by an independent commission backed by the United Nations, and other organizations including Physicians for Human Rights Israel and the Israeli human rights organization B'Tselem.^2,13–16^ Despite the assessments of genocide and the remarkably high toll that the Israeli Defense Force’s actions have had on children and other civilians,^6,17^ Israel’s campaign in Gaza has been supported militarily and politically by democratic countries that are the major funders of global health programmes and research. This in and of itself should compel those of us engaged in global health to speak out on this conflict, specifically. The ceasefire has done little to alleviate the humanitarian crisis.^18^

The silence of global health and medical professional organizations with respect to the genocide in Gaza implicitly condones efforts to delegitimize human rights and ethically motivated criticisms of Israel’s actions.^19^ On October 25 2025, the World Medical Association passed a resolution calling on the Israeli government to comply with the Geneva Conventions and other applicable instruments of humanitarian law.^20^ All global health and medical professional organizations need to do the same. We ask Union members to join us in calling on the Union to unite with other health and medical organizations in issuing a direct statement to address this specific, ongoing moral catastrophe.

What is needed? We advocate for:

- Cessation of hostilities by all parties and respect for the ceasefire agreement;^7,21^
- An end to Israel’s blockade of food, medical supplies and relief workers and a guarantee of unimpeded entry of medical and relief aid and coordinated diplomatic action by governments to ensure compliance with these obligations;
- Release of all health care workers currently imprisoned by Israel;
- Entry of independent international monitors to ensure the full application of protections afforded to healthcare facilities and workers in the Geneva Conventions;
- International collaboration to enable urgent evacuation of Palestinians in need of specialized care to health facilities in other countries, as needed, until the health system is rebuilt;
- Funding a Palestinian-led reconstruction of health, water and sanitation infrastructure in Gaza, ensuring community ownership and sustainability;^22^
- Funded training opportunities for Palestinians in medicine, nursing, allied health, and public health.

Finally, we express solidarity with healthcare workers in Gaza and everywhere under threat. We support similar advocacy efforts that specifically address human rights violations targeting healthcare workers and healthcare systems occurring elsewhere in the world. Defending medical ethics and international law is inseparable from the mission of global health itself.
